# Subclinical Brain Lesions in Magnetic Resonance Imaging are a Potential Indicator of Patent Foramen Ovale Related Migraines in Younger Patients

**DOI:** 10.31083/RCM37480

**Published:** 2025-08-29

**Authors:** Hong Yang, Fei Ma, Rui Li, Qiang Zhou, Fan Lin, Hesong Zeng, Dao Wen Wang, Jiangang Jiang, Xiang Luo, Hong Wang

**Affiliations:** ^1^Division of Cardiology and Department of Internal Medicine, Tongji Hospital, Tongji Medical College of Huazhong University of Science and Technology, 430030 Wuhan, Hubei, China; ^2^Hubei Key Laboratory of Genetics and Molecular Mechanism of Cardiologic Disorders, Huazhong University of Science and Technology, 430030 Wuhan, Hubei, China; ^3^Division of Neurology, Tongji Hospital, Tongji Medical College, Huazhong University of Science and Technology, 430030 Wuhan, Hubei, China

**Keywords:** patent foramen ovale, migraine, subclinical brain lesion, diffusion-weighted imaging

## Abstract

**Background:**

The causal relationship between migraines and patent foramen ovale (PFO) remains controversial, and a major unresolved question is how to define migraines attributable to PFO. Thus, this study aimed to determine if brain lesions could be a potential indicator of PFO-related migraines.

**Methods:**

Consecutive migraine patients from 2017 to 2019 who underwent transthoracic echocardiography or transcranial Doppler examination with an agitated saline contrast injection were assessed for right-to-left shunts. We then presented diffusion-weighted imaging (DWI) in brain magnetic resonance imaging and its association with PFO in the included patients.

**Results:**

A total of 424 patients with a mean age of 44.39 ± 12.06 years were included in this retrospective study. Among them, 244 patients (57.5%) had PFO, and 246 patients (58%) had subclinical brain lesions—the brain lesions presented as single or multiple scattered lesions. No association was observed between PFO prevalence and brain lesions in the total cohort (odds ratio (OR) 0.499); however, a significant association was observed in patients aged less than 46 years (OR, 3.614 in the group aged <34 years, 95% confidence interval (CI) 1.128–11.580, and 3.132 in the group of 34 years ≤ age < 46 years, 95% CI 1.334–7.350, respectively). Lesions in patients with PFO observed using DWI came more from the anterior or multiple than the posterior vascular territory (*p* = 0.033). DWI lesion numbers, location, and right-to-left shunt amounts did not affect the association between DWI-observed lesions and PFO.

**Conclusions:**

This study demonstrated that subclinical brain lesions are associated with PFO and may be used as a potential predictor of PFO-related migraines in patients aged less than 46 years. This may help identify candidate patients for PFO closure in future clinical decisions.

## 1. Introduction

Migraine is a disease with high prevalence and a high disability rate [1]. Despite intensive investigations in the past decades, its fundamental pathophysiology hasn’t been fully understood and the current therapies are often unsatisfactory [2, 3].

PFO was initially linked to migraine by the findings that the incidence of PFO was significantly higher in migraine patients than in the healthy controls, and in turn, patients with PFO suffer from migraine more frequently than the general population [4, 5, 6]. Subsequently, some observational studies reported that migraine patients responded well to PFO closure, which further strengthened the link between PFO and migraine [7, 8, 9]. However, all previous randomized controlled studies (RCTs) evaluating the benefits of PFO closure for migraine failed to reach the primary efficacy endpoint [10, 11, 12]. While a pooled analysis of occluder device trials has shown that the closure of PFO significantly reduces the frequency of migraine attacks and enables a greater number of subjects to complete migraine cessation, particularly in migraine with aura patients who are more likely PFO-associated migraine [13]. Therefore, PFO may be “incidental” or “causal”, these “incidental” PFO alone does not fully account for migraine attacks and guide the therapy of PFO closure in migraine. While those “causal” PFO relieves migraines after the PFO is turned off, as in PFO-related stroke [14]. How to determine the causal or incidental nature of the PFO and further guiding the closure treatment of PFO remain major challenges for future trials and clinical practice.

The characteristics of migraine patients who are more likely to be associated with PFO have been investigated. Several studies have shown a closer relationship between PFO and migraine with aura, especially atypical aura, than that without aura [15, 16, 17]. The higher attack frequency, headache impact test-6 (HIT-6), and migraine disability assessment (MIDAS) scores may also provide more evidence suggesting the relationship of PFO with migraine [18]. In turn, permanent PFO and PFO with large right to left shunt (RLS) may increase the incidence of migraine [19, 20], indicating that the migraine mechanism involving PFO more often results in aura symptoms. Although these clues, more objective and reliable predictors are required to help in the diagnosis of “true” PFO-related migraine.

Paradoxical tiny embolism is the most probable underlying mechanism of how PFO can cause migraine attacks, which may lead to tiny brain infarctions at the same time [21]. The effectiveness of antiplatelet and anticoagulation therapy in treating migraine provides evidence supporting this hypothesis [22, 23]. Furthermore, previous studies have shown that PFO-associated cryptogenic ischemic stroke patients are more common in young people, with an age range of approximately 32 to 42 years old [24, 25, 26]. Therefore, we hypothesized that the DWI imaging features of patients could indicate the presence of “true” PFO-related migraine in an age-dependent manner. In this study, the DWI features of migraine patients and their association with RLS amounts were investigated. We aimed to evaluate if the DWI pattern could be used to define the group of patients with probable “true” PFO-related migraine, which may assist in identifying candidate patients for PFO closure in future clinical decisions.

## 2. Methods

### 2.1 Study Population

Consecutive migraine patients <60 years old with or without aura who were referred to Tongji Hospital (Wuhan, China) and underwent either a transthoracic echocardiography (TTE) (n = 372, 372/424 = 87.7%) or a transcranial doppler (TCD) (n = 52, 52/424 = 12.3%) examination with agitated saline contrast (ASC) injection for assessment of PFO, from January 1, 2017 to December 31, 2019, were retrospectively screened. Among this cohort, we excluded the patients fulfilling the following criteria: (1) age ≥60 years; (2) had a history of stroke; (3) non-vascular causes of brain lesions; (4) had no brain magnetic resonance imaging (MRI) data; (5) with cardiac diseases including atrial fibrillation, congenital heart disease except PFO, heart failure, and significant valvular heart disease. Finally, a total of 424 patients were included in this study. The study was approved by the Institutional Review Board of the Ethics Committee of Tongji Hospital (TJ-IRB20230356) and was conducted by the ethical standards laid down in the 1964 Declaration of Helsinki and its later amendments. All participants provided written informed consent.

### 2.2 Brain MRI Study

Brain MRI DWI study was conducted for all enrolled patients and the results were analyzed by two independent neurologists who were blinded to the patient’s clinical information. A consensus was achieved in case of discrepancies. We evaluated the DWI lesion numbers, involved vascular territories, and DWI lesion locations. We first classified DWI lesions into three categories according to the lesion numbers: (1) no lesion; (2) single lesion; and (3) multiple lesions. We then assessed DWI lesions based on their vascular territory involvements and their locations. The vascular territories were: (1) posterior territory; and (2) anterior or multiple territories. DWI lesion locations were: (1) non-cortical lesion; (2) cortical lesion. The patients with cortical lesions were defined as patients who had cortical lesions only or had both cortical and non-cortical lesions.

### 2.3 TTE and TCD Study With ASC Injection 

TTE is a widely accessible and reliable technique for detecting PFO-related RLS [27] and thus served as the primary diagnostic tool for RLS in this study. TCD demonstrates greater sensitivity than TTE for detecting RLS during regular breathing. Consequently, for patients who did not undergo TTE examination, TCD examinations were considered. TTE or TCD was performed following intravenous ASC bolus injection during the Valsalva maneuver and at rest. For ASC bolus injection, a mixture of 8 mL saline with 1 mL of air and 1 mL of blood was agitated between two 10 mL syringes connected by a 3-way stopcock and then quickly injected into the brachial vein. Valsalva maneuver was completed by a forced expiration against a manometer to 40 mmHg [28].

TTE was performed using a Vivid E9 apparatus (GE Vingmed; Horten, Norway), and the apical four-chamber view was used for the visualization of microbubbles. At least one movie of the apical four-chamber view for resting and two movies for provocation were recorded. If a patient had a suboptimal TTE image or inconclusive RLS, we additionally injected ASC and acquired movies once more. To define the timing of RLS, the number of cardiac cycles when early microbubbles started to be seen in the left ventricular (LV) after right atrial (RA) opacification was counted. Early appearance (within three cardiac cycles) of microbubbles was defined as a positive RLS. Late appearance (after three cardiac cycles) was defined as an indeterminate shunt. TCD was carried out using a TCD monitoring device. Bilateral middle cerebral arteries were simultaneously monitored through the temporal window to detect microembolic signals (MESs) after ASC injection. MESs were recorded by computer software and counted by seeking high-intensity transient signals (HITS). TTE and TCD findings were analyzed by an expert who was blinded to the patient’s clinical data.

### 2.4 Definitions for RLS Amount

We analyzed the amount and diagnosis of RLS using both resting and provocation images. The amount of RLS was semi-quantified according to a 6-level scale modified from a previously described scale method [9]: 0 = absence of shunt (no microbubble or indeterminate shunt on TTE or TCD); 1 = latent shunt of mild degree (1–20 microbubbles after Valsalva maneuver on TTE or TCD); 2 = latent shunt of moderate degree (21–50 microbubbles after Valsalva maneuver on TTE or TCD); 3 = latent shunt of high degree (>50 microbubbles on TTE or curtain on TCD after Valsalva maneuver); 4 = permanent shunt of mild/moderate degree (>10 microbubbles at rest and >50 microbubbles on TTE or curtain on TCD after Valsalva maneuver); 5 = (>50 microbubbles on TTE and curtain on TCD at rest). RLS were then classified as (1) no RLS (scale 0); (2) small RLS (scale 1–2); and (3) large RLS (scale 3–5). Patients with the presence of RLS, either small or large, were diagnosed as PFO in the study.

### 2.5 Statistical Analysis

Categorical variables were expressed as frequencies (percentages), normally distributed continuous data as mean with standard deviation (SD). Non-normally distributed continuous data are presented as the median with interquartile range (IQR). The normality of distribution was assessed using the Kolmogorov-Smirnov test. Continuous variables between groups were analyzed by the Student t-test or Mann-Whitney U test according to the normality of the variables. Categorical variables were analyzed using the Chi-square test or Fisher’s exact test, as appropriate. To avoid multicollinearity, variance inflation factor (VIF) and tolerance were used to evaluate covariates and eliminated variables with a VIF over 5. Univariate logistic regression analysis was performed to identify clinically relevant variables associated with the prevalence of PFO in the total cohort and DWI lesions in the PFO cohort. Multivariable logistic regression models further adjusted age, sex, hypertension, diabetes, dyslipidemia, and current smoking according to clinical, the correlation is identified by using a backward stepwise selection. Subgroup analyses were performed based on age or with or without RLS to confirm the associations between PFO prevalence and DWI lesions (positive and negative), DWI brain lesions and the RLS amounts. Intra- and interobserver reproducibility of MRI parameters was assessed by calculating the intra-class correlation coefficients (ICCs) on 20 randomly selected patients. All analyses were conducted using R software (R Foundation for Statistical Computing, version 4.2.0, Vienna, Austria) and GraphPad Prism (GraphPad Prism Software Inc., version 9.0, San Diego, CA, USA). Statistical tests were two-tailed, and a *p* value of <0.05 was considered statistically significant.

## 3. Results

### 3.1 Demographic, Clinical, and Brain Imaging Characteristics Between Migraine Patients With and Without PFO 

The demographic, clinical, and brain imaging characteristics were compared in migraine patients with and without PFO and described in Table 1. The study included a total of 424 patients with migraine for analysis, of whom 244 patients (57.5%) had PFO. The mean age was 44.39 ± 12.06 years. There were more females (62.7%) in the patient cohort, but there was no sex difference between patients with and without PFO. The other clinical characteristics, including hypertension, diabetes, dyslipidemia, and current smoking, were also not different between patients with and without PFO.

**Table 1.** **Different demographic, clinical, and brain imaging characteristics between patients with and without PFO**.

Variables		Total (n = 424)	PFO-negative (n = 180)	PFO-positive (n = 244)	*p*-value
Female, n (%)		266 (62.7)	112 (62.2)	154 (63.1)	0.851
Age, years (mean ± SD)		44.39 ± 12.06	45.81 ± 13.04	43.34 ± 11.22	0.089
Hypertension, n (%)		117 (27.6)	51 (28.3)	66 (27.0)	0.771
Diabetes, n (%)		48 (11.3)	19 (10.6)	29 (11.9)	0.670
Dyslipidemia, n (%)		127 (30.0)	56 (31.1)	71 (29.1)	0.656
Current smoking, n (%)		61 (14.4)	27 (15.0)	34 (13.9)	0.758
DWI brain lesion, n (%)					0.379
	Negative	178 (42.0)	80 (44.4)	98 (40.2)	
	Positive	246 (58.0)	100 (55.6)	146 (59.8)	
DWI brain lesion positive, n (%)		Total (n = 246)	PFO-negative (n = 100)	PFO-positive (n = 146)	*p*-value
DWI brain lesion number, n (%)					0.065
	Single	40 (16.3)	11 (11.0)	29 (19.9)	
	Multiple	206 (83.7)	89 (89.0)	117 (80.1)	
Involved vascular territory of brain lesion, n (%)					0.033
	Anterior or multiple	213 (86.6)	81 (81.0)	132 (90.4)	
	Posterior	33 (13.4)	19 (19.0)	14 (9.6)	
Brain lesion location, n (%)					0.476
	Non-cortical lesion	30 (12.2)	14 (14.0)	16 (11.0)	
	Cortical lesion	216 (87.8)	86 (86.0)	130 (89.0)	

Data are mean with SD for continuous variables or frequencies (%) for categorized variables; *p*-value calculated for PFO-positive vs PFO-negative; the group of patients with cortical lesion included patients who had cortical lesion only and patients who had both cortical and non-cortical lesion. PFO, patent foramen ovale; DWI, diffusion-weighted imaging.

Of 424 patients, 246 (58%) had DWI lesion. Among them, 16.3% had single lesion and 83.7% had multiple lesions. They all presented as small scattered lesion, of which 87.8% were cortical lesion (cortical only or both cortical and non-cortical lesion) and 86.6% were from the anterior vascular territory. In the analyses that compared the difference in DWI pattern between patients with and without PFO, the presence or absence of DWI lesion, lesion number, and lesion location (cortical or non-cortical) were all not associated with the presence or absence of PFO. However, the proportion of patients with single DWI lesion was higher among PFO-positive than among PFO-negative (19.9% vs 11.0%). Moreover, DWI lesion was more likely to come from anterior or multiple vascular territories instead of posterior vascular territory in PFO-positive patients compared with PFO-negative patients (*p* = 0.033) (Table 1).

### 3.2 DWI Lesion and Its Association With PFO Presence 

Multivariable logistic regression analysis revealed no association between the PFO prevalence and the DWI brain lesion (OR 0.499, 95% confidence interval [CI] 0.236–1.052, *p* = 0.286) (**Supplementary Table 1**). We then tested the association between DWI lesion and PFO presence in different age groups (Table 2). All patients were divided into four groups based on age quartiles: (1) age <34 years; (2) 34 years ≤ age < 46 years; (3) 46 years ≤ age < 55 years; (4) age ≥55 years. DWI lesion-positive was found in 246/424 patients (58%) and the incidence of DWI lesion-positive increased with age (from 9.3% in the group of age <34 years to 41.9% in the group of age ≥55 years).

**Table 2.** **PFO prevalence in patients with or without DWI brain lesions according to different age groups**.

DWI lesion-negative, n (%)		Total (n = 178)	PFO-negative (n = 80)	PFO-positive (n = 98)	*p*-value
	age <34	81 (45.5)	35 (43.8)	46 (46.9)	0.673
	34 ≤ age < 46	56 (31.5)	27 (33.8)	29 (29.6)	0.555
	46 ≤ age < 55	30 (16.8)	13 (16.2)	17 (17.4)	0.713
	age ≥55	11 (6.2)	5 (6.2)	6 (6.1)	0.972
DWI lesion-positive, n (%)		Total (n = 246)	PFO-negative (n = 100)	PFO-positive (n = 146)	*p*-value
	age <34	23 (9.3)	4 (4.0)	19 (13.0)	0.017
	34 ≤ age < 46	48 (19.5)	11 (11.0)	37 (25.3)	0.005
	46 ≤ age < 55	72 (29.3)	30 (30.0)	42 (28.8)	0.835
	age ≥55	103 (41.9)	55 (55.0)	48 (32.9)	<0.001

Data are frequencies (%) for categorized variables; *p*-value calculated for PFO-positive vs. PFO-negative.

**Fig. 1.**
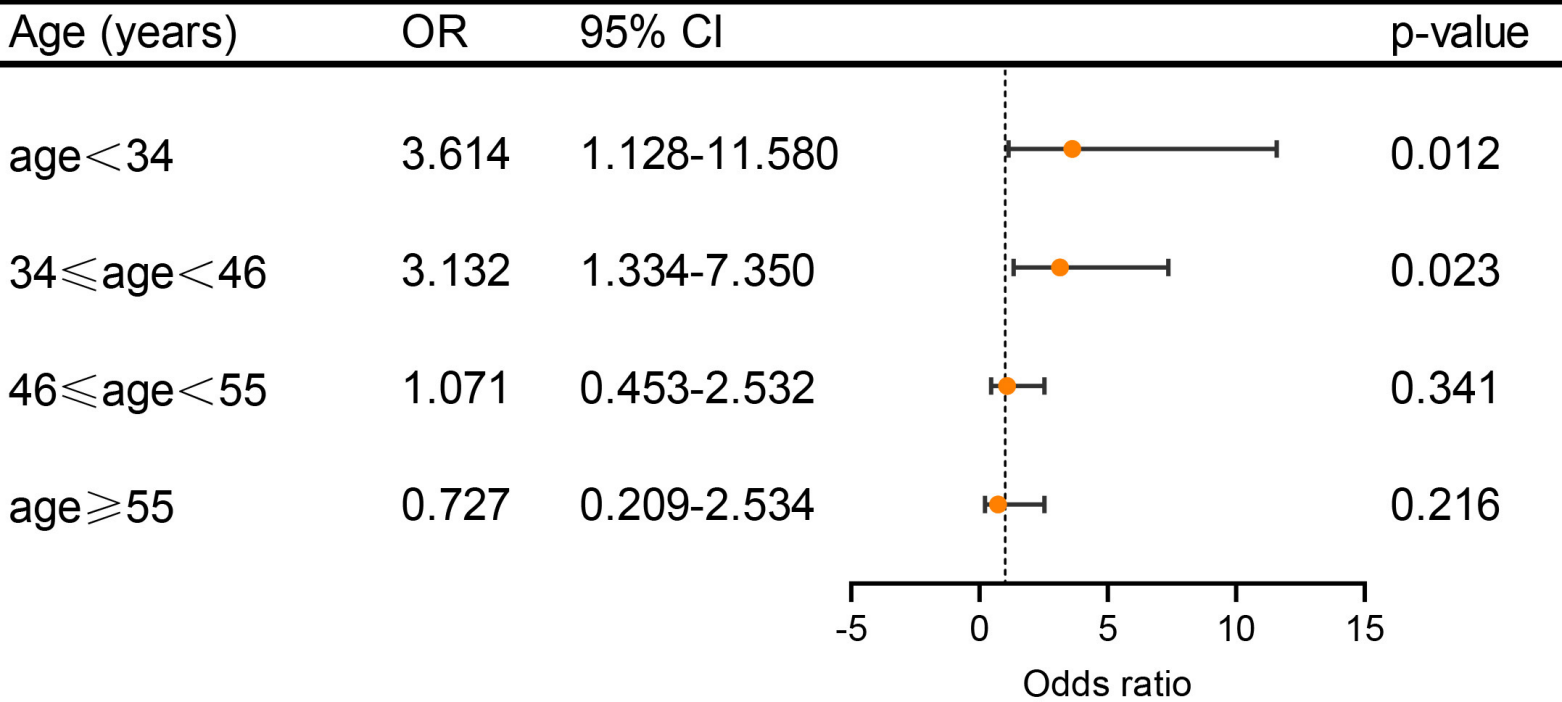
**The association between PFO prevalence and DWI lesions (positive and negative) under different age subgroups in the total cohort**. Adjusted ORs (including sex, hypertension, diabetes, dyslipidemia, and current smoking) and 95% CIs of PFO prevalence were determined using logistic regression analysis. With the increase of age, the risk of PFO prevalence in patients with DWI lesion positive gradually decreases. PFO, patent foramen ovale; DWI, diffusion-weighted imaging; OR, odds ratio; CI, confidence interval.

In DWI lesion-positive patients, the proportion of PFO presence was significantly higher than that of PFO absence in the group of patients who were younger than 46 years (13% vs 4%, *p* = 0.017, in the group of age <34 years; and 25.3% vs 11%, *p* = 0.005, in the group of 34 years ≤ age < 46 years, respectively). In turn, in the group of 46 years ≤ age < 55 years, the proportion of PFO-positive and PFO-negative patients was not significantly different (28.8% vs 30%, *p* = 0.835). Interestingly, in the group of age ≥55 years, the proportion of PFO-positive patients was inversely lower than that of PFO-negative patients (32.9% vs 55%, *p* < 0.001). Subsequently, the association between PFO prevalence and DWI lesions (positive and negative) in different age subgroups was explored in the total cohort, adjusted ORs (including sex, hypertension, diabetes, dyslipidemia, and current smoking), and 95% CIs of PFO prevalence were determined using logistic regression analysis, which revealed that the odds of PFO presence in patients with DWI lesion-positive were diminished by older age (Fig. 1). The OR value was 3.614 (95% CI 1.128–11.580, *p* = 0.012) in group of age <34 years, 3.132 (95% CI 1.334–7.350, *p* = 0.023) in group of 34 years ≤ age < 46 years, 1.071 (95% CI 0.453–2.532, *p* = 0.341) in 46 years ≤ age < 55 years and 0.727 (95% CI 0.209–2.534, *p* = 0.216) in group of age ≥55. In comparison, in DWI lesion-negative patients, there was no difference between the proportion of PFO-negative and PFO-positive patients for all four age groups (Table 2).

These findings suggest that the association of DWI lesion with the presence of PFO in migraine patients is age-dependent. The association is significant only in patients younger than 46 years but not in those equal to or older than 46 years.

### 3.3 DWI Lesion and Its Association With RLS Amounts 

**Fig. 2.**
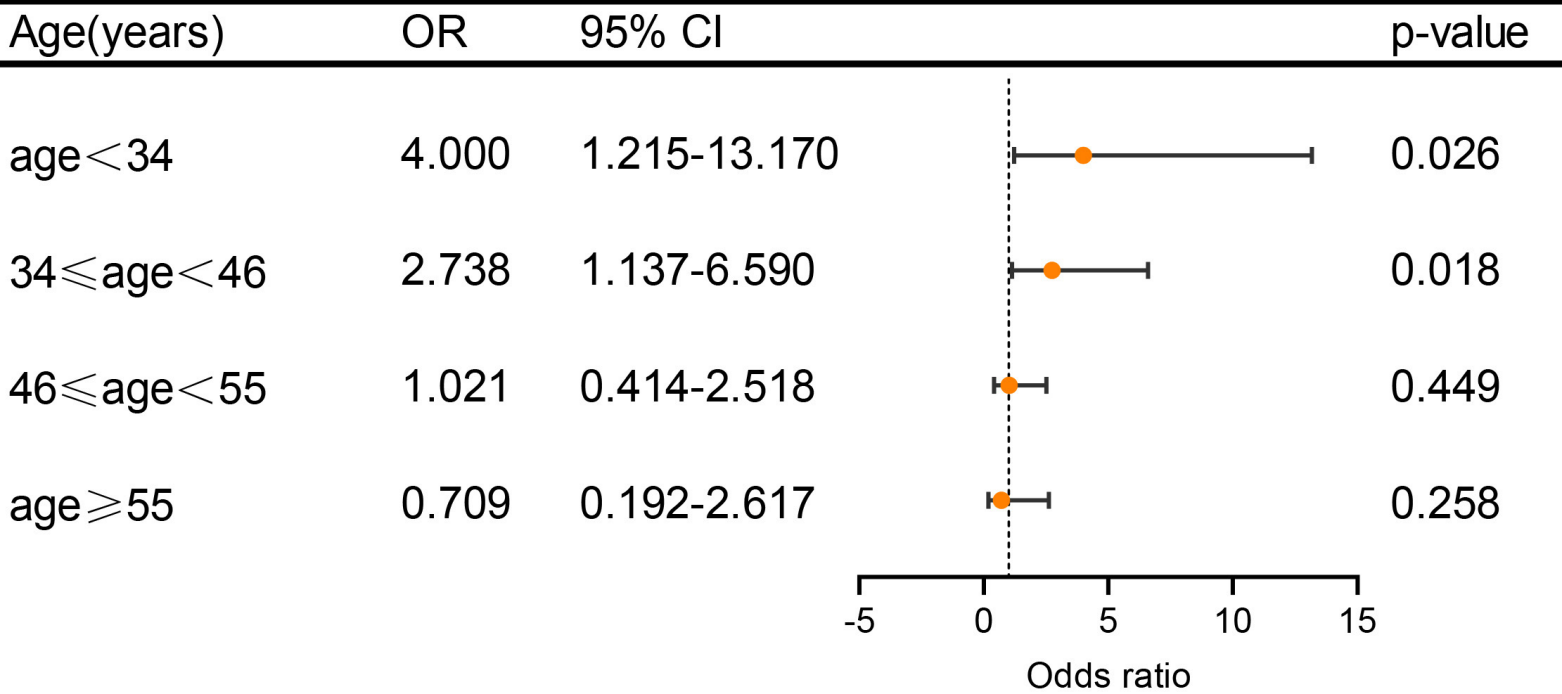
**The association between DWI lesions (positive and negative) and PFO with large RLS amounts under different age subgroups in PFO-positive patients**. Adjusted ORs (including sex, hypertension, diabetes, dyslipidemia, and current smoking) and 95% CIs of DWI lesions-positive were determined using logistic regression analysis. With the increase of age, the prevalence of DWI lesion-positive gradually decreases. RLS, right to left shunt. Other abbreviations as in Fig. 1.

**Fig. 3.**
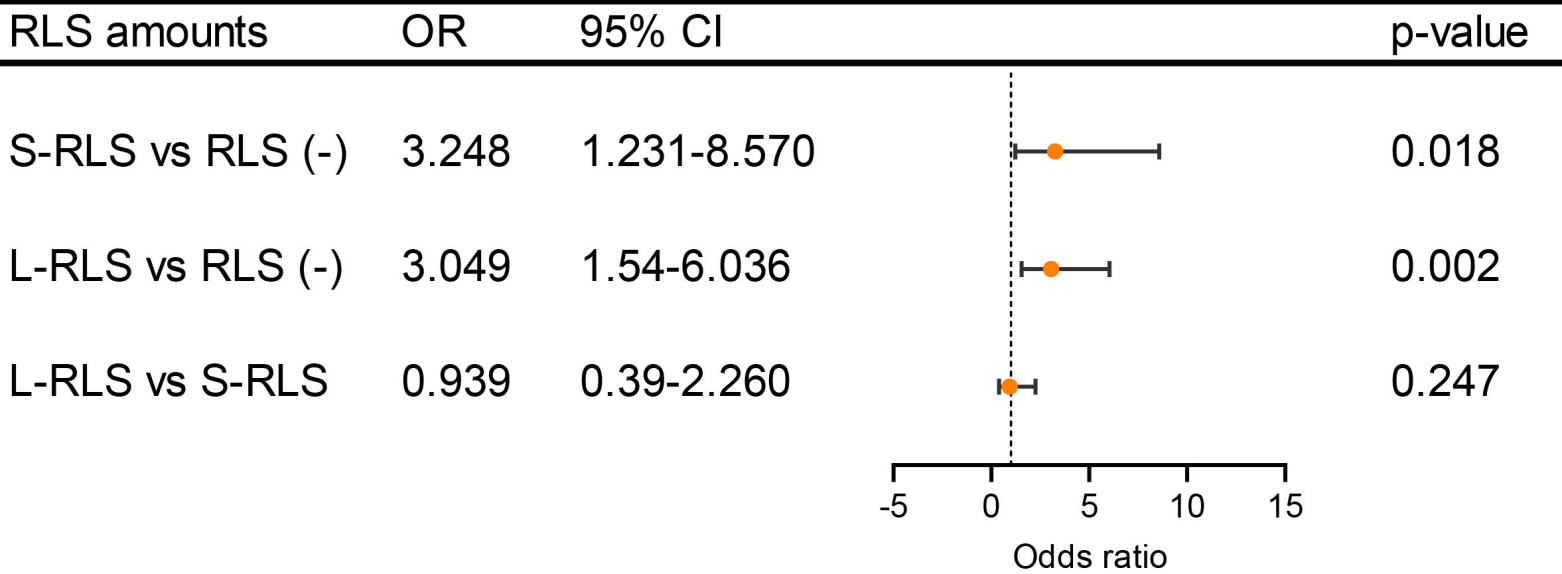
**The association of DWI brain lesion in patients younger than 46 years according to different RLS amounts**. There was no difference in the association of DWI brain lesion between L-RLS vs S-RLS (OR 0.939, *p* = 0.247). While compared with RLS (-), L-RLS (OR 3.049, *p* = 0.002) and S-RLS (OR 3.248, *p* = 0.018) are associated with DWI brain lesion. L-RLS, large right to left shunt; S-RLS, small right to left shunt; RLS (-), right to left shunt negative. Other abbreviations as in Figs. 1,2.

We tested the association between brain DWI lesion and RLS amounts. In 244 patients (57.5%) who had PFO, the RLS amounts were distributed as (1) 47 (19.3%) with small RLS and (2) 197 (80.7%) with large RLS. Multivariable logistic regression analysis found no association between RLS amounts and the DWI brain lesions (positive and negative) (OR 1.020, 95% CI 0.993–1.047, *p* = 0.248) (**Supplementary Table 2**). However, we further tested the association between DWI lesions (positive and negative) and PFO with large RLS amounts under different age subgroups in PFO-positive patients, adjusted ORs (including sex, hypertension, diabetes, dyslipidemia, and current smoking) and 95% CIs of DWI lesions-positive were determined using logistic regression analysis, found that the prevalence of DWI lesion-positive in patients with PFO with large RLS amounts were also age dependent and diminished by older age (Fig. 2). The OR value was 4.000 (95% CI 1.215–13.170, *p* = 0.026) in the group of age <34 years, 2.738 (95% CI 1.137–6.590, *p* = 0.018) in the group of 34 years ≤ age < 46 years, 1.021 (95% CI 0.414–2.518, *p* = 0.449) in 46 years ≤ age < 55 years and 0.709 (95% CI 0.192–2.617, *p* = 0.258) in the group of age ≥55. To confirm whether DWI brain lesion is related to the RLS amounts, subgroup analyses based on with or without RLS in patients who were aged less than 46 years, adjusted ORs (including sex, hypertension, diabetes, dyslipidemia, and current smoking) and 95% CIs of DWI lesions were determined using logistic regression analysis, there was no difference for the prevalence of brain lesion between large-RLS and small-RLS (OR 0.939, 95% CI 0.390–2.260, *p* = 0.247) (Fig. 3). In comparison, OR was 3.049 (95% CI 1.540–6.036, *p* = 0.002) between large-RLS and RLS negative, and 3.248 (95% CI 1.231–8.570, *p* = 0.018) between small-RLS and RLS negative (Fig. 3). The results revealed that the association between the PFO and DWI lesion is probably independent of RLS amounts in migraine patients.

### 3.4 Reproducibility

Intra-observer agreement analysis showed an ICC of 0.99 (95% CI 0.99–1.00, *p* < 0.001) for DWI brain lesion, of 0.97 (95% CI 0.94–0.99, *p* < 0.001) for DWI brain lesion number, of 0.95 (95% CI 0.82–0.98, *p* < 0.001) for involved vascular territory of brain lesion, and of 0.98 (95% CI 0.92–0.99, *p* < 0.001) for brain lesion location, showing excellent agreement. Inter-observer agreement analysis showed an ICC of 0.95 (95% CI 0.82–0.98, *p* < 0.001) for DWI brain lesion, of 0.94 (95% CI 0.82–0.98, *p* < 0.001) for DWI brain lesion number, of 0.90 (95% CI 0.88–0.92, *p* < 0.001) for involved vascular territory of brain lesion, and of 0.93 (95% CI 0.83–0.97, *p* < 0.001) for brain lesion location, indicating a good performance level.

## 4. Discussion

This study found that a subgroup of migraineurs showed characteristic DWI patterns, single or multiple small scattered lesions. The association of DWI lesion with PFO presence is probably age-dependent and the prevalence of PFO was much higher in patients with DWI lesion who were younger than 46 years. The findings strongly suggest that PFO presence is one of the underlying etiologies leading to subclinical brain lesion in migraine, particularly in younger migraineurs. Therefore, DWI lesion from the brain MRI could be supporting evidence of PFO-related migraine in younger migraineurs and could aid in identifying candidate patients for PFO closure in future clinical decisions.

In migraine patients, numerous studies reported the incidence of PFO was 14.6–66.5% in comparison with 9–27.3% in the general population [5, 21, 29]. In this study, PFO was found in 57.5% of migraine patients, which is consistently higher than that in the general population and in line with previous studies. Numerous studies also linked migraine with an increased risk of stroke [30, 31], and brain lesions were frequently observed at MRI in patients with migraine according to previous studies [32, 33]. In our study, asymptomatic DWI lesion was found in 58% of migraine patients, which is remarkably higher than 7.2–17.7% in the general population [34, 35]. However, the precise mechanism underlying the link between migraine and stroke remains unclear. Whether PFO is one of the shared causal factors by both migraine and stroke has not been confirmed. Notably, some studies have found that the presence of PFO in migraineurs is more prevalent among females, approximately 14.6–58.6% [24, 25, 36, 37]. While the present study found that the prevalence of PFO was 63.1% in females and 36.9% in males. Moreover, among migraine patients with or without PFO, females as a specific risk factor for cryptogenic ischemic stroke [25], potentially attributable to hormonal influences, particularly fluctuations in estrogen levels [38]. When the mechanism involves RLS, certain blood components typically cleared or reduced in the pulmonary vasculature, such as 5-hydroxytryptamine (5-HT), may reach cerebral circulation through the PFO at abnormal concentrations, thereby triggering migraines [14].

A single infarction or multiple small-scattered lesions in the brain have been reported to be the typical imaging pattern of a PFO-related stroke [39, 40, 41], and included in paradoxical embolism (RoPE) score to detect PFO-attributable cryptogenic stroke [42]. Considering paradoxical embolism as the common underlying mechanism for PFO-stroke and PFO-migraine, we hypothesized that small brain lesions may also provide supporting evidence for PFO-related migraine. However, in the whole cohort of the study, we didn’t reveal the association between the PFO prevalence and the presence of brain lesion. An early study has proved the importance of age in determining the association between PFO and cryptogenic stroke [43]. Age has been incorporated into the RoPE score to stratify the probability of PFO-attributable cryptogenic stroke [42]. Interestingly, the association between migraine and ischemic stroke is stronger in women younger than 45 years [44]. Therefore, we performed the analysis by incorporating the age factor. In the study cohort of Mas *et al*. [24], PFO-associated cryptogenic ischemic stroke patient’s mean age was 41.9 years, and 58.6% were women. Similarly, Putaala *et al*.’s study [25] revealed that the incidence of young-onset ischemic stroke is rising, ischemic stroke with PFO patient’s mean age was 40 years, and 37 ± 5 years in Vigna *et al*.’s study [26]. In our study, the results proved a significantly strong correlation between PFO and brain lesion in patients who were aged less than 46 years. The correlation was diminished with older age and even reversed in patients older than 55 years. In older patients, vascular disease and vascular risk factors such as hypertension are more likely the major reasons causing the ischemic brain lesions. In younger patients who are less likely to have vascular disease, PFO is probably the major underlying factor for the ischemic lesion by paradoxical embolism. PFO with RLS may increase the incidence of migraine [19, 20], and the prevalence of migraine increased with increasing volume of RLS (37.9% for small RLS, 46.7% for moderate RLS, and 49.4% for large RLS) [45]. On the contrary, patients with and without aura of migraine are also more likely to have RLS, the prevalence was 63.8% in the migraine with aura and 39.9% in the migraine without aura [46]. PFO with massive RLS has been recognized as a high-risk PFO and aids clinical decisions in selecting patients with cryptogenic stroke for PFO closure [41, 47]. However, RLS amounts didn’t correlate with either brain lesion presence or lesion numbers in our study, which might be due to the decline in statistical power caused by a small sample size. Further study is needed to evaluate the significance of RLS amounts in PFO-related migraine. Overall, our results proved the importance of age in determining the association of DWI lesion with PFO in migraine and suggested the potential role of subclinical brain lesion as an indicative of PFO-related migraine in patients who are aged less than 46 years, which may help identify candidate patients with PFO closure in future clinical decisions.

We also compared the DWI features in migraine patients with PFO and without PFO in the study, including the lesion location, number, and involved vascular territory. The imaging characteristics of the brain lesions in migraine patients with PFO were very similar to those reported in PFO-related stroke [39, 40, 41], such as single or small scattered lesions and multiple vascular territory involvement. Brain lesion numbers didn’t differ significantly between PFO-positive and PFO-negative patients. However, the prevalence of single brain lesions was higher in patients with PFO. As to the involved vascular territory, the brain lesions of the patients with PFO in the study were more likely to come from anterior or multiple territories. The predominance of either posterior or anterior circulation involvement in PFO-related stroke has been reported in previous studies [41, 48] and the significance of specific vascular territory involvement in defining PFO-related migraine needs further studies. Brain lesion in PFO-related stroke is more likely superficial or cortical. In contrast, no difference was found between cortical and non-cortical lesion in the study. The cortical lesion in our study was defined as multiple lesions that were both cortical and non-cortical, which may affect the results. Whether the location of brain lesion is useful in defining PFO-related migraine needs further studies as well.

## 5. Limitations

There are several limitations in the study. First, the study was a retrospective single-center study with a relatively small size. Thus, patient selection bias should be considered. For example, some patients were pre-selected in the local hospital and then referred to our program, which will probably cause higher PFO incidence in the study population. Second, the results from the study can only demonstrate the relationship between PFO and migraine with brain lesions but can’t confirm the causality. Prospective studies, especially studies testing the efficacy of PFO closure in migraineurs with cortical lesions, are needed. Third, the diagnostic method used to detect PFO may influence the study results. Although the current gold standard for evaluating PFO is transesophageal echocardiography (TEE), it has not been used as the first-line screening tool for PFO in clinical practice. This study primarily utilized TTE as a diagnosis tool, however, the diagnostic performance of TTE for PFO may be compromised by artifacts originating from the chest wall. Similarly, TCD is limited by the availability of a good cranial window for ultrasounds and by the impossibility of determining the RLS anatomical location. Nevertheless, the accuracy of TTE and TCD with ASC injection in diagnosing PFO has been confirmed in previous studies [49, 50, 51, 52, 53] and is recommended by the guidelines [27, 54]. These two modalities were used in diagnosing PFO in our study cohort, of whom 52 (12.3%) patients were diagnosed with TCD. Since TTE and TCD exhibit differing levels of diagnostic accuracy, the number of microbubbles required for diagnostic yield and for distinguishing large versus small shunts may vary between these two modalities, it may introduce a certain degree of error in RLS diagnosis and classification. Fourth, adequate Valsalva maneuver which is important to detect PFO was not assessed by objective findings as decreased E velocity in the study, although the Valsalva maneuver against a manometer to 40 mmHg has been frequently used in clinical practice and its sensitivity to detect RLS has been proved [28]. Fifth, the baseline data lack biological markers, resulting in an insufficient description of the baseline characteristics. Sixth, in subgroup analysis by age, the Type I error may increase with the multiple comparisons. Finally, other confounding factors contributing to the relationship between PFO and migraine, such as migraine type, migraine attack frequency, PFO size, and underlying vascular disease, are not considered in the study and may also bias our results.

## 6. Conclusion

The debate about the implication of PFO as an etiology for migraine and the benefit of PFO closure for treating migraine has been around for a few decades. Although previous RCTs evaluating the benefits of PFO closure for migraine failed to obtain positive results, a recent clinical study has shown that closing the PFO significantly reduces the frequency of migraine attacks and even complete migraine cessation in PFO-associated migraine. However, selecting patients with PFO-associated migraine remains a major challenge for the design of future trials and clinical decision-making. Although it is very difficult to find direct evidence in PFO-related migraineurs, our study demonstrated that the presence of brain lesions and younger age are consistently associated with increasing prevalence of PFO. We proposed that the presence of subclinical brain lesions is probably valuable in determining the probability of PFO-related migraine and the value is age-dependent. The subgroup of migraine patients with PFO who are aged less than 46 years and at the same time have subclinical brain lesions by MRI could be true PFO-related migraineurs and the appropriate candidates for PFO closure.

## Data Availability

The datasets generated during and analyzed during the current study are available from the corresponding author upon reasonable request.

## Abbreviations

ASC, agitated saline contrast; DWI, diffusion-weighted imaging; HITS, high-intensity transient signal; MESs, microembolic signals; RCTs, randomized controlled studies; MRI, magnetic resonance imaging; PFO, patent foramen ovale; RLS, right-to-left shunt; RoPE score, paradoxical embolism score; TCD, transcranial doppler; TTE, transthoracic echocardiography; TEE, transesophageal echocardiography.

## Supplementary Material

Supplementary material associated with this article can be found, in the online version, at https://doi.org/10.31083/RCM37480.
