# Supplementary material for: Subclinical Brain Lesions in Magnetic Resonance Imaging are a Potential Indicator of Patent Foramen Ovale Related Migraines in Younger Patients
Source: Rev Cardiovasc Med. 2025 Aug 29;26(8):37480. doi: 10.31083/RCM37480 (PMC12415766; doi:10.31083/RCM37480)
Supplement: Supplementary file 1 [file 2153-8174-26-8-37480-s1.docx]

**Supplementary Table 1**

Univariate and multivariate logistic regression analysis of predictors of PFO prevalence in the total cohort.

| Variables | Univariate | | Multivariate | |
| --- | --- | --- | --- | --- |
|  | OR (95% CI) | p- value | OR (95% CI) | p- value |
| Age | 1.026 (0.999-1.053) | 0.072 | 1.020 (0.970-1.060) | 0.272 |
| Female | 1.391 (0.733-2.640) | 0.216 | 1.150 (0.593-2.228) | 0.314 |
| Hypertension | 1.080 (0.973-1.199) | 0.104 | 1.035 (0.993-1.079) | 0.115 |
| Diabetes | 1.142 (0.941-1.392) | 0.412 | 1.017 (0.962-1.075) | 0.541 |
| Dyslipidemia | 1.060 (0.960-1.170) | 0.204 | 1.124 (0.916-1.380) | 0.262 |
| Current smoking | 1.013 (0.990-1.031) | 0.190 | 1.008 (0.988-1.028) | 0.268 |
| DWI lesion | 0.855 (0.354-2.060) | 0.203 | 0.499 (0.236-1.052) | 0.286 |

Model adjusted for sex, hypertension, diabetes, dyslipidemia, and current of smoking. PFO: patent foramen ovale; OR: odds ratio; CI: confidence interval; DWI: diffusion weighted imaging.

**Supplementary Table 2**

Univariate and multivariate logistic regression analysis of predictors of DWI lesions in the PFO patients.

| Variables | Univariate | | Multivariate | |
| --- | --- | --- | --- | --- |
|  | OR (95% CI) | p- value | OR (95% CI) | p- value |
| Age | 1.033 (0.965-1.105) | 0.145 | 1.024 (0.983-1.066) | 0.252 |
| Female | 1.171 (0.975-1.406) | 0.326 | 1.123 (0.782-1.613) | 0.529 |
| Hypertension | 1.069 (0.789-1.450) | 0.338 | 1.016 (0.900-1.147) | 0.547 |
| Diabetes | 1.063 (0.963-1.172) | 0.352 | 1.020 (0.948-1.099) | 0.595 |
| Dyslipidemia | 1.124 (0.987-1.063) | 0.143 | 1.073 (0.967-1.191) | 0.182 |
| Current of smoking | 1.001 (0.993-1.002) | 0.210 | 1.011 (0.997-1.026) | 0.123 |
| RLS amounts | 1.313 (0.770-2.241) | 0.218 | 1.020 (0.993-1.047) | 0.348 |

Model adjusted for sex, hypertension, diabetes, dyslipidemia, and current smoking. PFO: patent foramen ovale; OR: odds ratio; CI: confidence interval; DWI: diffusion weighted imaging; RLS: right to left shunt.
